# Fibroblast growth factor receptor splice variants are stable markers of oncogenic transforming growth factor β1 signaling in metastatic breast cancers

**DOI:** 10.1186/bcr3623

**Published:** 2014-03-11

**Authors:** Michael K Wendt, Molly A Taylor, Barbara J Schiemann, Khalid Sossey-Alaoui, William P Schiemann

**Affiliations:** 1 Department of Medicinal Chemistry and Molecular Pharmacology, Purdue University, Hansen Life Sciences Building, 201 S University Street, West Lafayette, IN 47907, USA; 2 Case Comprehensive Cancer Center, Case Western Reserve University, Wolstein Research Building, 2103 Cornell Road, Cleveland, OH 44106, USA; 3 Department of Molecular Cardiology, Lerner Research Institute, Cleveland Clinic, 9500 Euclid Avenue, Cleveland, OH 44195, USA

## Abstract

**Introduction:**

Epithelial–mesenchymal transition (EMT) and mesenchymal–epithelial transition (MET) facilitate breast cancer (BC) metastasis; however, stable molecular changes that result as a consequence of these processes remain poorly defined. Therefore, with the hope of targeting unique aspects of metastatic tumor outgrowth, we sought to identify molecular markers that could identify tumor cells that had completed the EMT:MET cycle.

**Methods:**

An *in vivo* reporter system for epithelial cadherin (E-cad) expression was used to quantify its regulation in metastatic BC cells during primary and metastatic tumor growth. Exogenous addition of transforming growth factor β1 (TGF-β1) was used to induce EMT in an *in situ* model of BC. Microarray analysis was employed to examine gene expression changes in cells chronically treated with and withdrawn from TGF-β1, thus completing one full EMT:MET cycle. Changes in fibroblast growth factor receptor type 1 (FGFR1) isoform expression were validated using PCR analyses of patient-derived tumor tissues versus matched normal tissues. *FGFR1* gene expression was manipulated using short hairpin RNA depletion and cDNA rescue. Preclinical pharmacological inhibition of FGFR kinase was employed using the orally available compound BGJ-398.

**Results:**

Metastatic BC cells undergo spontaneous downregulation of E-cad during primary tumor growth, and its expression subsequently returns following initiation of metastatic outgrowth. Exogenous exposure to TGF-β1 was sufficient to drive the metastasis of an otherwise *in situ* model of BC and was similarly associated with a depletion and return of E-cad expression during metastatic progression. BC cells treated and withdrawn from TGF-β stably upregulate a truncated FGFR1-β splice variant that lacks the outermost extracellular immunoglobulin domain. Identification of this FGFR1 splice variant was verified in metastatic human BC cell lines and patient-derived tumor samples. Expression of FGFR1-β was also dominant in a model of metastatic outgrowth where depletion of FGFR1 and pharmacologic inhibition of FGFR kinase activity both inhibited pulmonary tumor outgrowth. Highlighting the dichotomous nature of FGFR splice variants and recombinant expression of full-length FGFR1-α also blocked pulmonary tumor outgrowth.

**Conclusion:**

The results of our study strongly suggest that FGFR1-β is required for the pulmonary outgrowth of metastatic BC. Moreover, FGFR1 isoform expression can be used as a predictive biomarker for therapeutic application of its kinase inhibitors.

## Introduction

The reported results from several recent studies suggest that metastatic breast cancer (BC) cells undergo epithelial–mesenchymal transition (EMT) during invasion and dissemination and that the reverse process of mesenchymal–epithelial transition (MET) occurs at some point during metastatic tumor outgrowth [1-3]. In fact, the ability of BCs to transition between an epithelial and mesenchymal state seems to be a key feature of the metastatic process and has recently been more accurately termed *epithelial–mesenchymal plasticity*[4]. Indeed, many of the well-established changes in gene expression that take place during EMT return to baseline during MET. However, stable phenotypic markers capable of distinguishing metastatic BC cells that have undergone an EMT:MET cycle from their indolent counterparts that have not undergone an EMT program remain to be identified.

Recently, we demonstrated that transforming growth factor β (TGF-β)–induced EMT empowers BCs with the ability to invade in response to paracrine epidermal growth factor (EGF) stimulation, thereby facilitating the egress of BC cells from the primary tumor [5]. Intriguingly, we also observed that cells proficient in undergoing metastatic outgrowth downregulate EGF receptor (EGFR) during the MET process [2]. These findings may serve to explain the contrasting clinical data that establish EGFR as a predictor of poor prognosis for BC patients [6], yet administration of monotherapies directed against EGFR or in conjunction with other chemotherapies has failed to provide a clinical benefit for BC patients [7-9].

Increased expression of fibroblast growth factor receptor (FGFR) types 1 and 3 have recently been identified as two of six receptor tyrosine kinases associated with poor disease-free survival and/or decreased overall survival in BC patients [10]. FGFR1, FGFR2 and FGFR3 all exist as several different isoforms generated via alternative splicing [11]. Two of the best-described variants are generated via inclusion of unique versions of the third (III) extracellular immunoglobulin (III-Ig) domain, and hence they are termed *FGFR-IIIb* and *FGFR-IIIc*[11]. The III-Ig domain governs the specificity of FGFR binding to the 18 different FGF ligands. FGF2 (basic FGF), for instance, has an extremely high affinity for the IIIc isoform [12]. Another FGFR splicing event results in either the inclusion (FGFR-α) or exclusion (FGFR-β) of the first Ig domain and/or the linker region between IgI and IgII, an area termed the *acid box*[13]. Importantly, IgI and the linker region regulate the affinity of FGFR for its particular ligand [14]. Furthermore, increased expression of the β versus α isoform of FGFR1 has been correlated with reduced relapse-free survival in a cohort of BC patients [15]. The use of antisense morpholino oligonucleotides to convert FGFR splicing from the β to the α isoform can induce apoptosis in glioblastomas [16]. Currently, the upstream mechanisms that regulate FGFRα/FGFRβ splicing remain poorly defined, but TGF-β and its induction of EMT can cause upregulation of FGFR1-IIIc and downregulation of FGFR2-IIIb [17]. However, little is known about the mechanism by which either of these events drives the metastatic progression of BCs.

In The present study, we sought to identify factors that are stably altered during EMT:MET cycles and thus might act as drivers of metastatic tumor outgrowth. To this end, we utilized microarray expression analyses of BC cells that had been treated and withdrawn from exogenous TGF-β to uncover a stable upregulation of the FGFR1-β isoform, an event that was also readily detected in samples obtained from BC patients and in metastatic human BC cell lines. Along these lines, genetic depletion of total FGFR1 and/or ectopic overexpression of FGFR1-α in the D2.A1 model of pulmonary metastatic outgrowth potently inhibited pulmonary tumor formation. Collectively, these studies establish FGFR1-β as a critical player whose expression is stably altered in metastatic BCs that have experienced oncogenic TGF-β signaling and undergone EMT:MET cycles. Our findings highlight the need to further elucidate the pro- and antitumorigenic nature of FGFR to appropriately administer small-molecule inhibitors for the treatment of metastatic BC.

## Methods

### Cell lines and reagents

Murine D2.A1 and human MCF-10A derivatives (MCF-10A, T1K and Ca1h) were ethically obtained from Dr Fred Miller (Wayne State University, Detroit, MI, USA) [18,19], and murine 4T1, human MCF-7 and human MDA-MB-231 cells were purchased from the American Type Culture Collection (ATCC; Manassas, VA, USA). In addition, the following human BC cell lines were originally purchased from the ATCC and ethically obtained from Dr John J Pink (Case Western Reserve University, Cleveland, OH, USA): MDA-MB-361, ZR-75-1, T47D, T47D-C42W and BT549 [20]. The aforementioned human and murine BC cells were cultured in Dulbecco’s modified Eagle’s medium supplemented with 10% fetal bovine serum and 1% penicillin-streptomycin as described previously [5]. Bioluminescent 4T1 and D2.A1 cells were engineered to stably express firefly luciferase under the selection of Zeocin (InvivoGen, San Diego, CA, USA) as described previously [2,5,21]. Bioluminescent normal mammary epithelial (NME) cells were constructed and cultured as previously described [5]. Dual-bioluminescent E-cad reporter cells were generated by stably transfecting 4T1 cells with pcDNA3.1/Hygro mammalian expression vector (Invitrogen, Carlsbad, CA, USA) encoding Renilla luciferase under control of the cytomegalovirus (CMV) promoter and pGL4.20[*luc2*/Puro] vector (Promega, Madison, WI, USA) that encodes firefly luciferase under control of the human *Cdh1* promoter [22]. Cellular depletion of FGFR1 expression was achieved by glycoprotein of vesicular stomatitis virus lentiviral transduction of TRC pLKO.1 short hairpin RNA (shRNA) vectors (Thermo Scientific, Pittsburgh, PA, USA) (Additional file 1: Table S1) as described previously [2,21]. Ectopic expression of FGFR1-α-IIIc was accomplished as described previously and selected for using neomycin [2].

### *In vivo* bioluminescence imaging of tumor growth and metastasis

Parental (that is, scrambled shRNA) and FGFR1-manipulated D2.A1 cells were injected into the lateral tail veins of 5-week-old female BALB/C mice (The Jackson Laboratory, Bar Harbor, ME, USA). Where indicated, mice bearing D2.A1 pulmonary tumors were treated daily with BGJ-398 (ChemieTek, Indianapolis, IN, USA) or PF-573271 (PF271; Pfizer Pharmaceuticals, New York, NY, USA) at 50 mg/kg by oral gavage. Alternatively, *Cdh1* reporter 4T1 cells (1 × 10^4^ cells) were engrafted onto the mammary fat pads of 4-week-old BALB/c mice. Circulating 4T1 tumor cells were isolated from the inferior vena cava at the time of necropsy using 3% sodium citrate. Following lysis of red blood cells, circulating tumor cells were selected for with 5 μg/ml Zeocin (the selectable marker for firefly luciferase). Luciferase-expressing NME cells (1 to 2 × 10^6^ cells) were engrafted onto the mammary fat pads of 5-week-old female *nu*/*nu* mice. All bioluminescent images were captured using a Xenogen IVIS 200 preclinical imaging system (Caliper Life Sciences/PerkinElmer, Hopkinton, MA, USA) within the Small Animal Imaging Resource Center at the Case Comprehensive Cancer Center as previously described [5,21,23].

### Gene expression profiling

NME cells were cultured in the presence of TGF-β1 (5 ng/ml) for 4 weeks, at the end of which TGF-β1 supplementation was discontinued and the cells were allowed to recover for an additional 4 weeks. Total RNA was prepared from unstimulated cells of similar passage (pre-TGF) and the post-TGF NME cells. Microarray analyses were performed in triplicate using the GeneChip Mouse Gene ST 1.0 Array (Affymetrix, Santa Clara, CA, USA). Genes regulated more than twofold are given in Additional file 2: Table S2. The complete data set has been deposited in the National Center for Biotechnology Information Gene Expression Omnibus (GEO) database [GEO:GSE54491] [24].

### mRNA transcript analyses

For real-time PCR analysis, normal murine mammary gland (NMuMG) and NME cells were stimulated with TGF-β1 (5 ng/ml) for varying lengths of time, and then total RNA was isolated using RNeasy Plus Mini Kit (QIAGEN, Valencia, CA, USA). Afterward, total RNA was reverse-transcribed using the iScript cDNA Synthesis Kit (Bio-Rad Laboratories, Hercules, CA, USA), and semiquantitative real-time PCR was conducted using iQ SYBR Green Supermix (Bio-Rad Laboratories) as described previously [21]. Identification of FGFR splice variants was accomplished by visualizing PCR products separated by gel electrophoresis. The oligonucleotide primer pairs used are provided in Additional file 1: Table S1.

### Immunoblotting and immunohistochemical analyses

For immunoblot assays, lysates generated from two- and three-dimensional cultures were prepared as described previously [2]. The antibodies we used are described in Additional file 3: Table S3. For immunohistochemistry, tissues were fixed in 10% formalin, and histological sections were prepared by the Tissue Procurement and Histology Core at the Case Comprehensive Cancer Center. Sections were deparaffinized and stained with the indicated antibodies given in Additional file 3: Table S3.

### Three-dimensional organotypic growth assays

Cells were diluted in complete media supplemented with 5% Cultrex reagent (Trevigen, Gaithersburg, MD, USA) and seeded onto solidified Cultrex cushions (50 μl/well) contained in 96-well plates (1 × 10^4^ cells/cm^2^). Longitudinal bioluminescence growth assays were performed as described previously [2,5]. Pharmacological inhibitors against FGFR (BGJ-398) or focal adhesion kinase (FAK) (PF271) were added to cultures at the indicated concentrations and times.

### Study approval

All animal procedures were performed in accordance with protocols approved by the Institutional Animal Care and Use Committee of the Case Western Reserve University School of Medicine. For human BC specimens, primary tumors and matched normal tissues were collected and processed under protocols approved by the Institutional Review Board of the Cleveland Clinic. All patients provided their written informed consent allowing the study investigators to have access to their tumor specimens and clinical data.

### Statistical analyses

Statistical values were defined using an unpaired Student’s *t*-test, where a *P*-value less than 0.05 was considered significant. Statistically significant differences in the overall survival of mice bearing control and FGFR-manipulated D2.A1 pulmonary tumors were analyzed using a logrank test. *P* values for all experiments are indicated.

## Results

### E-cadherin is dynamically regulated during spontaneous breast cancer metastasis

**Figure 1:**
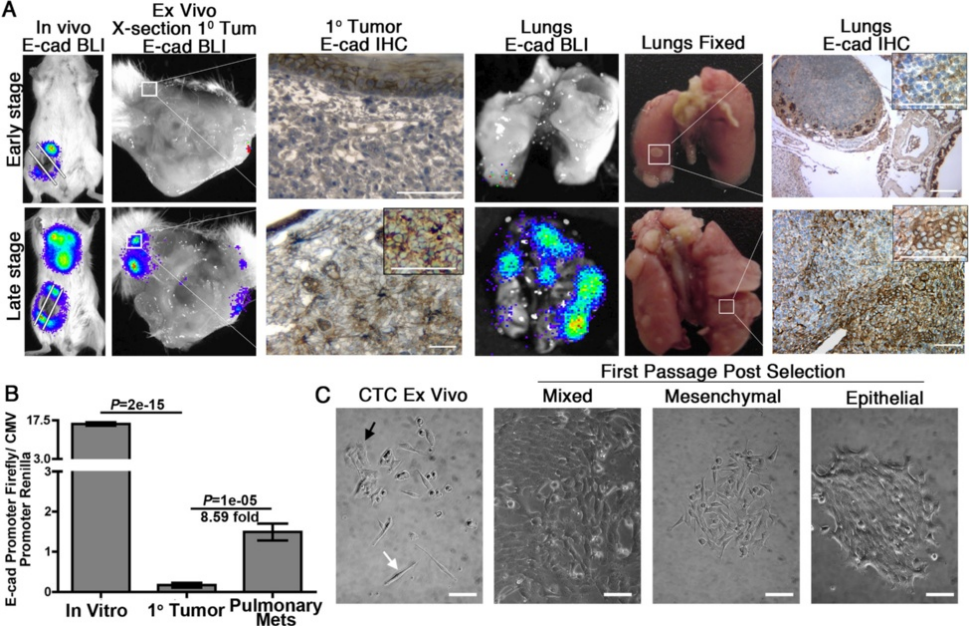
**Spontaneously metastatic breast cancer cells dynamically regulate epithelial cadherin during *in vivo *primary and metastatic tumor growth. (A)** Bioluminescence imaging (BLI) of metastatic 4T1 cells expressing cytomegalovirus (CMV)-driven Renilla luciferase and firefly luciferase driven by the epithelial cadherin (E-cad) promoter. Cells were engrafted onto the mammary fat pads of BALB/c mice, and qualitative E-cad promoter activity (for example, luciferin-derived bioluminescence) was monitored *in vivo* and *ex vivo*. E-cad bioluminescence was spatially correlated with E-cad protein expression as determined by IHC. Bars indicate 40x, 100x and 400x magnifications. **(B)** E-cad promoter activity (for example, luciferin-derived bioluminescence) was quantified by normalization to CMV promoter activity (for example, coelenterazine-derived bioluminescence) in *in vitro* cultured cells and *ex vivo* tissues derived from primary tumors and their late-stage metastases (Mets). Data represent ten samples (n = 10, ±SE) derived from five individual mice bearing primary and metastatic tumors, resulting in the indicated *P* values. **(C)** Circulating tumor cells (CTCs) were isolated from the blood of 4T1 tumor-bearing mice and selected for resistance to Zeocin. Photomicrographs show the antibiotic-resistant CTCs (CTC *ex vivo*). The morphologically epithelial cells (black arrow) were physically isolated from the mesenchymal cells (white arrow) and further subcultured as separate populations of mesenchymal-like and epithelial-like cells as indicated. Bars indicate a 100x magnification.

We recently established that BCs that can downregulate E-cadherin (E-cad) are at a selective advantage to initiate metastatic outgrowth within the pulmonary microenvironment [2,25]. However, these and other studies suggest that, as pulmonary metastases progress from micro- to macroscopic lesions, E-cad expression returns in a manner reminiscent of the differentiation status observed in noninvasive primary tumors [1,2]. Along these lines, the highly metastatic 4T1 model of late-stage BC displays several epithelial characteristics, including the expression of E-cad [2,21]. Therefore, we sought to use this model system in combination with dual-substrate bioluminescence [26] to track the *in vivo* dynamics of E-cad regulation as 4T1 cells progress through the metastatic cascade. To this end, 4T1 cells that stably expressed Renilla luciferase under the control of the CMV promoter were engineered to stably express firefly luciferase under the control of the E-cad promoter [2]. Using this approach, we observed areas within primary tumors that failed to express E-cad luminescence (Figure 1A). Large areas in the center of the primary tumor were necrotic and therefore lacked luminescence, so we utilized immunohistochemistry (IHC) to verify a lack of E-cad protein in several outer and viable areas of the tumor (Figure 1A). Furthermore, early nodular metastases produced little to no firefly luminescence, and they were similarly verified by IHC analysis to be viable tumor tissue and E-cad-negative (Figure 1A). Once these metastatic lesions had spread deeper into the pulmonary tissue, however, E-cad luminescence and protein returned in a manner consistent with that of a MET program (Figure 1A). To verify that the differences in firefly bioluminescence were due to regulation of the E-cad promoter, primary and metastatic tumors were homogenized and the firefly luminescence was normalized to the CMV-driven expression of Renilla luciferase expressed in the same cells. This quantitative approach resulted in an 8.59-fold upregulation of E-cad promoter activity in pulmonary metastases as compared to their corresponding primary tumors (Figure 1B). Consistent with the role of EMT in facilitating primary tumor dissemination, we readily captured highly mesenchymal-appearing, antibiotic-resistant tumor cells from the peripheral blood of mice bearing primary 4T1 tumors (Figure 1C). When left as a general cell population, however, the circulating tumor cells quickly resumed an epithelial phenotype in culture (Figure 1C). Taken together, the results of these studies suggest that the ability to efficiently transit between epithelial and mesenchymal states is associated with efficient completion of the metastatic cascade.

### TGF-β treatment is sufficient to drive orthotopic mammary tumor metastasis

**Figure 2:**
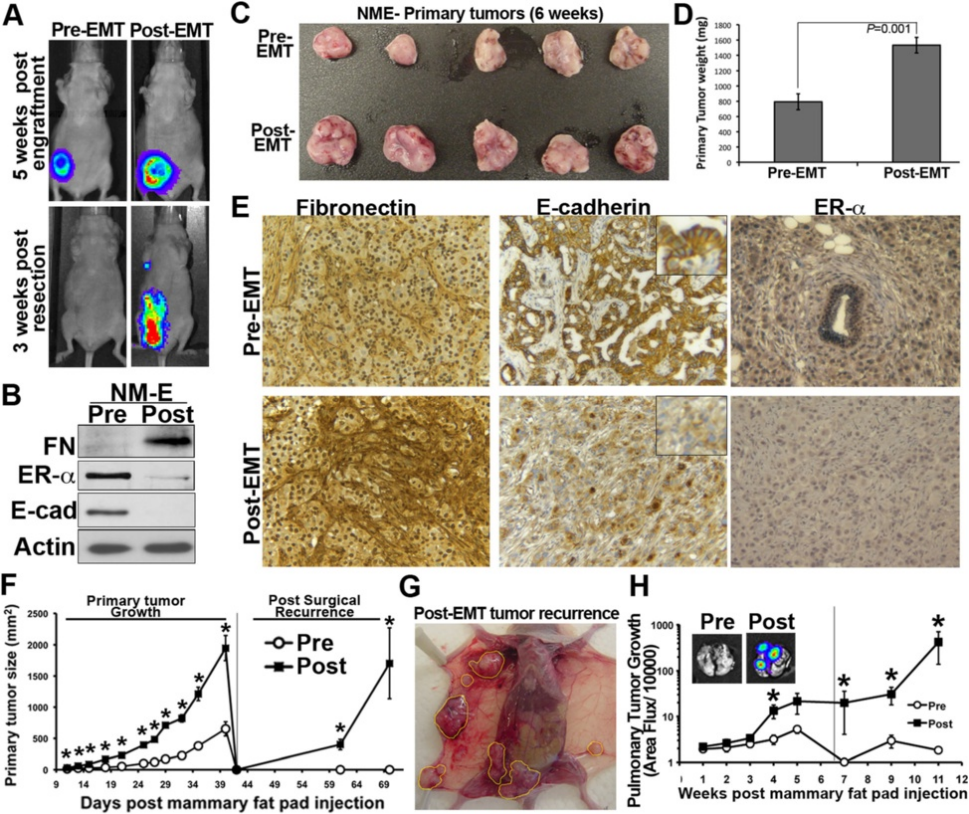
**Transforming growth factor β treatment is sufficient to induce postsurgical recurrence and pulmonary metastasis. (A)** Normal mammary epithelial (NME) cells were left untreated (before transforming growth factor treatment (pre-TGF)) or treated with TGF-β1 (post-TGF) and engrafted (2 × 10^6^ cells/mouse) onto the mammary fat pad, at which point primary tumor growth was monitored by bioluminescence. Five weeks after engraftment of pre- and post-TGF NME cells, the primary tumors were surgically removed and tumor recurrence was similarly monitored by bioluminescence imaging. Shown are two representative mice from each experimental cohort, both before and 3 weeks after surgical resection of the primary tumor. **(B)** NME cells were treated with TGF-β1 (Post) as described in (A) and monitored for expression of the mesenchymal marker fibronectin (FN) and the marker epithelial cadherin (E-cad). Estrogen receptor α (ERα) was potently downregulated in NME cells following TGF-β treatment. **(C)** Photograph showing *ex vivo* pre- and post-TGF primary tumors following surgical resection demonstrating complete and intact primary tumor excision. **(D)** The average weight (±SE) of pre- and post-TGF primary tumors shown in (C) and the indicated *P* value. **(E)** Pre- and post-TGF primary tumors were analyzed by immunohistochemistry for the *in vitro* markers described in Panel B. All images were taken at 100x magnification and insets were taken at 400x magnification. **(F)** Growth of pre- and post-TGF NME primary and recurrent tumors were monitored using digital caliper measurements (n=5 mice/group). Vertical line indicates time of primary tumor resection. Data are the mean (±SE) tumor size for each group . *P < 0.05. **(G)** Photograph of a representative mouse 3 weeks after resection of a post-TGF NME tumor. Yellow outlines show widespread recurrent tumor formation. As shown in (A), mice bearing pre-TGF NME tumors failed to form recurrent tumors. **(H)**  Pulmonary metastasis of pre- and post-TGF NME tumors was quantified by bioluminescence. Line indicates time of primary tumor resection. Inset: Bioluminescent images of *ex vivo* lungs from representative mice bearing pre- and post-TGF NME tumors. Data are the mean (±SE) area flux values for each group. *P < 0.05 (n = 5 mice/group).

TGF-β is one of several “master regulators” of the EMT process [27]. Given the spontaneous nature of E-cad regulation during 4T1 metastasis, we next sought to specifically address whether TGF-β treatment would be sufficient to facilitate the metastasis of primary orthotopic mammary tumors. To do so, we utilized our established model in which NMuMG cells are transformed via overexpression of EGFR (NME cells) [5]. Although orthotopic tumor formation in this model is robust, postsurgical recurrence and distant metastasis were not observed (Figure 2A). In stark contrast to control NME tumors, however, treatment of NME cells with TGF-β prior to mammary fat pad engraftment (Figure 2B) yielded larger primary tumors (Figures 2A to 2D) that displayed *in vivo* modulation of the EMT markers E-cad and fibronectin, as well as downregulation of estrogen receptor α (ER-α) (Figure 2E). More importantly, these TGF-β-treated tumors displayed aggressive recurrence following primary tumor resection (Figures 2A, 2F and 2G), and progressed to form pulmonary metastases in a fashion that was significantly increased as compared to the control tumors (Figure 2H). Taken together, these data clearly establish that TGF-β treatment is sufficient to induce the postsurgical recurrence and metastasis of an otherwise indolent model of *in situ* mammary carcinoma.

### FGFR is stably upregulated following TGF-β treatment and withdrawal

Given the transient nature of exogenous TGF-β treatment prior to NME cell engraftment, we next sought to assess the regulation of MET in this model. Consistent with our IHC analysis (Figure 2E), *ex vivo* subculture of post-EMT NME primary tumors displayed a highly mesenchymal phenotype in two- and three-dimensional cultures and downregulation of E-cad (Additional file 4: Figures S1A and S1B). TGF-β-treated NME tumor cells that had undergone metastasis were subcultured from the lungs of mice. In comparison to their parental NME counterparts, these cells underwent dramatically enhanced primary tumor formation, postsurgical recurrence and spontaneous pulmonary metastasis upon secondary mammary fat pad engraftment (Additional file 4: Figure S1C). These resulting metastases were subcultured and termed the *NME lung metastatic* (NME-LM2) cell line (Additional file 5: Figure S2A). Further *in vitro* analyses of the NME-LM cell lines revealed a return of E-cad expression to levels that approximated those detected in their untreated NME parental cells (Additional file 5: Figures S2B and S2C). Taken together, these results indicate that depletion of E-cad by exogenous TGF-β treatment was stably maintained during formation of the primary tumor but readily returned to baseline expression levels during formation of macroscopic pulmonary metastases.

Given that TGF-β treatment and metastasis are capable of selecting for a spontaneously metastatic cell model that has renewed E-cad expression, we next sought to identify stable changes in gene expression that could characterize cells that reside in either a pre- or post-TGF-β exposure state. To do so, we conducted microarray analyses of NME cells that had undergone 4 weeks of exogenous TGF-β1 treatment, which was followed by an additional 4-week withdrawal of exogenous TGF-β1 (Additional file 2: Table S2). Indeed, following this experimental protocol, the expression patterns of traditional EMT markers, such as E-cad, N-cadherin, vimentin, fibronectin, α-smooth muscle actin, Twist and Snail all returned to baseline levels (Additional file 2: Table S2). Surprisingly, 98 genes were stably modulated more than threefold following this TGF-β treatment and withdrawal protocol. Gene set enrichment analysis revealed that the highest degree of overlap between genes upregulated in our gene list was that of genes downregulated in mouse embryonic fibroblasts after 10 hours of TGF-β treatment [GEO:GSE15871] [28]. Furthermore, 15 upregulated and 28 downregulated genes in our data set were shared with a data set generated upon knockout of the TGF-β family member bone morphogenic protein 2 [29]. The transcription factor Snai2, which functions to inhibit the expression of E-cad [30], was downregulated tenfold in cells that had undergone this cycle of TGF-β treatment and withdrawal. Taken together, these findings suggest that certain aspects of TGF-β signaling can be stably altered, leading to a unique transcriptional profile in cells following prolonged ligand exposure and withdrawal.

**Figure 3:**
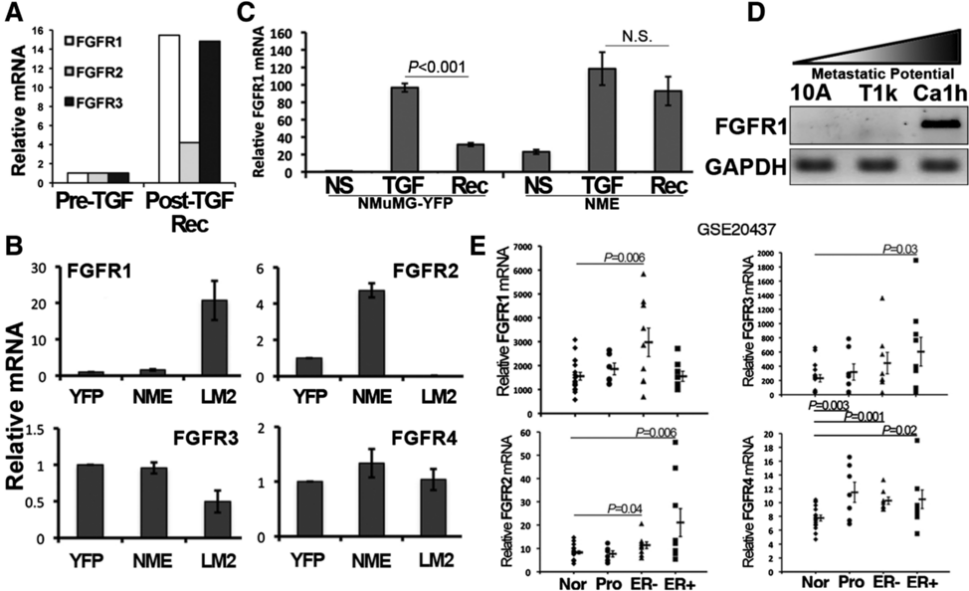
**Upregulation of fibroblast growth factor receptor type 1 is stably maintained during oncogenic transforming growth factor β signaling. (A)** Normal mammary epithelial (NME) cells were left untreated (before transforming growth factor treatment (pre-TGF)) or treated and allowed to recover from exogenous TGF-β1 treatment (post-TGF-Rec) as described in the Methods section. Global gene expression was assessed by microarray analyses. Data are results of RT-PCR analysis confirming stable upregulation of fibroblast growth factors 1 through 3 (FGFR1 to FGFR3) following TGF-β1 treatment and withdrawal. **(B)** Real-time PCR analyses showing the differential expression of FGFR1 to FGFR4 in control yellow fluorescent protein (YFP), nonmetastatic (NME) and lung metastatic (LM2) cell lines derived from the normal murine mammary gland (NMuMG) cells. **(C)** Control NMuMG (YFP) and NME cells were not stimulated (NS), stimulated with TGF-β1 (5 ng/ml) for 48 hours (TGF) and subsequently recovered for an additional 48 hours without exogenous ligand (Rec). Expression of FGFR1 was analyzed by real-time RT-PCR. Data in panels B and C are the mean expression values (±SD) of three independent experiments, resulting in the indicated *P*-values. **(D)**  Human MCF-10A-derived breast cancer (BC) cells of increasing metastatic potential were analyzed by RT-PCR for their expression of FGFR1. Glyceraldehyde 3-phosphate dehydrogenase (GAPDH) served as a loading control. Data are representative of three independent experiments. **(E)** *In silico* analyses of the Gene Expression Omnibus data set [GEO:GSE20437] demonstrating significant increases in FGFR1 to FGFR4 in prophylactically removed breast tissue (Pro), as well as in ER-negative (ER-) and ER-positive (ER+) tumor samples, as compared to a cohort of normal (Nor) breast tissue samples. Data are the individual values for each sample resulting in the indicated mean values (±SE) and *P* values.

Further analysis of the microarray data established that three EGFR ligands, betacellulin, amphiregulin and epiregulin were all potently downregulated (Additional file 6: Figure S3). Conversely, FGFR1, FGFR2 and FGFR3 were upregulated 15.8-, 4.2- and 14.8-fold, respectively (Figure 3A). Given these findings and a growing interest in targeting FGFR as a treatment for BC [31], we sought to further assess the role of FGFR in facilitating pulmonary metastatic outgrowth. In doing so, we first examined FGFR expression in our NME progression series. In this system generated *in vivo*, FGFR1 was the predominant family member stably upregulated in the metastatic NME-LM2 cells (Figure 3B). Using a condensed *in vitro* TGF-β treatment and withdrawal experiment, we demonstrate the stable upregulation of FGFR1 in the transformed NME cells as compared to their nontransformed NMuMG counterparts (Figure 3C). Similarly to the NME progression series, the human MCF10A BC progression series also displayed a robust increase in FGFR1 expression in cells with a higher metastatic potential (Figure 3D). Importantly, analysis of 20 human BC samples revealed a five- to tenfold increase in the expression of FGFR1 to FGFR4 in tumor tissue as compared to matched normal tissue (Additional file 7: Table S4). Furthermore, *in silico* analysis of the GEO data set [GEO:GSE20437] in which we compared normal and prophylactically removed mammary tissue to ER + and ER - tumor tissues demonstrated significant increases in several FGFR family members (Figure 3E). In light of other recent findings that suggest a role for FGFR1 in driving the progression of basal-type BCs [10,32-34], our findings clearly indicate that FGFR1 is upregulated in BCs with increasing metastatic potential. They also suggest that FGFR1 may be capable of driving the pulmonary outgrowth of metastatic BCs.

### FGFR1-β-IIIc is selected for during metastatic progression

**Figure 4:**
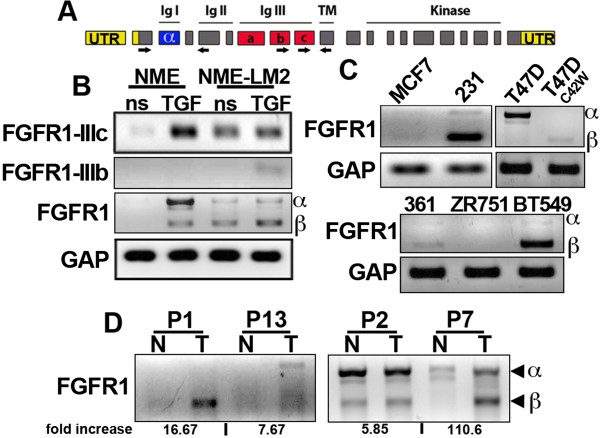
**The β isoform of the fibroblast growth factor receptor type 1 is selected for in increasingly metastatic cells and can be readily identified in patient tumor samples. (A)** Schematic representation the FGFR1 transcript depicting the coding regions for the immunoglobulin (Ig), transmembrane (TM), and kinase domains. The location of the unique IIIb/IIIc primer sets (arrows) and the flanking primer set detecting the inclusion or exclusion of the α exon (arrows) are also indicated. **(B)** Expression of FGFR1 isoforms depicted in (A) were analyzed by RT-PCR in the nonmetastatic normal mammary epithelial (NME) cells and their lung metastatic (LM) and isogenic NME-LM2 counterparts before (ns) and after a 48-hour treatment with 5 ng/ml transforming growth factor β (TGF). **(C)** Luminal MCF-7, T47D, MDA-MB-361 and ZR-75-1 and basal MDA-MB-231 and BT549 human breast cancer cells were analyzed for inclusion or exclusion of the α exon of FGFR1. The estrogen-independent T47D-C42W cells were also analyzed. Data in (B) and (C) are representative of three independent experiments. **(D)** Four of the patient tumor samples (T; patients 1, 2, 7 and 13 (P1, P2, P7 and P13, respectively) that demonstrated upregulation of FGFR1 as compared to their matched normal mammary tissues (N) (Additional file 7: Table S4) were further analyzed for expression of the α versus β isoforms of FGFR1. The fold increase in total FGFR1 expression as determined by real-time PCR analysis that was normalized to glyceraldehyde 3-phosphate dehydrogenase (GAP) is indicated.

TGF-β has recently been shown to be capable to modulating FGFR expression and alternative splicing of the FGFR1-IIIb/FGFR1-IIIc exon [17]*.* Indeed, examination of NME cells using isoform-specific RT-PCR primer sets (Figure 4A) indicated that TGF-β treatment upregulated the expression of FGFR1-IIIc (Figure 4B). Importantly, this modulated expression of FGFR1-IIIc was maintained throughout the metastatic process as NME-LM2 cells displayed constitutive upregulation of FGFR1-IIIc (Figure 4B). However, FGFR1 is subject to further alternative splicing that leads to the inclusion or exclusion of the third (α) exon that encodes the outermost Ig domain (IgI) of the receptor [12] (Figure 4A). Importantly, increased expression of the β isoform of FGFR1 has previously been associated with breast cancer [15]. Although inclusion of the α exon is dominant following *in vitro* TGF-β treatment of NME cells, it is the truncated β isoform that was selected for and enriched during the metastatic generation of the NME-LM2 cells (Figure 4B). Examination of several human BC cell lines revealed that the luminal MCF-7, T47D, MDA-MB-361 and ZR-75-1 cells all express little to no FGFR1-β (Figure 4C). In contrast, the basal-like and metastatic BT549 and MDA-MB-231 human BC cells demonstrate robust expression of the truncated FGFR1-β isoform (Figure 4C). Interestingly, examination of a T47D subline, C42W, that was selected for by prolonged estrogen deprivation [20] displayed a loss of the FGFR1-α isoform and a slight gain in FGFR1-β (Figure 4C). Furthermore, two of the four patient tumor samples that displayed increased FGFR1 expression as compared to their matched surrounding normal tissues (Additional file 7: Table S4) also demonstrated expression of the truncated FGFR1-β isoform (Figure 4D). Collectively, these results strongly suggest that expression of FGFR1-β is increased with metastatic progression.

### Genetic depletion of FGFR1 prevents pulmonary tumor outgrowth

**Figure 5:**
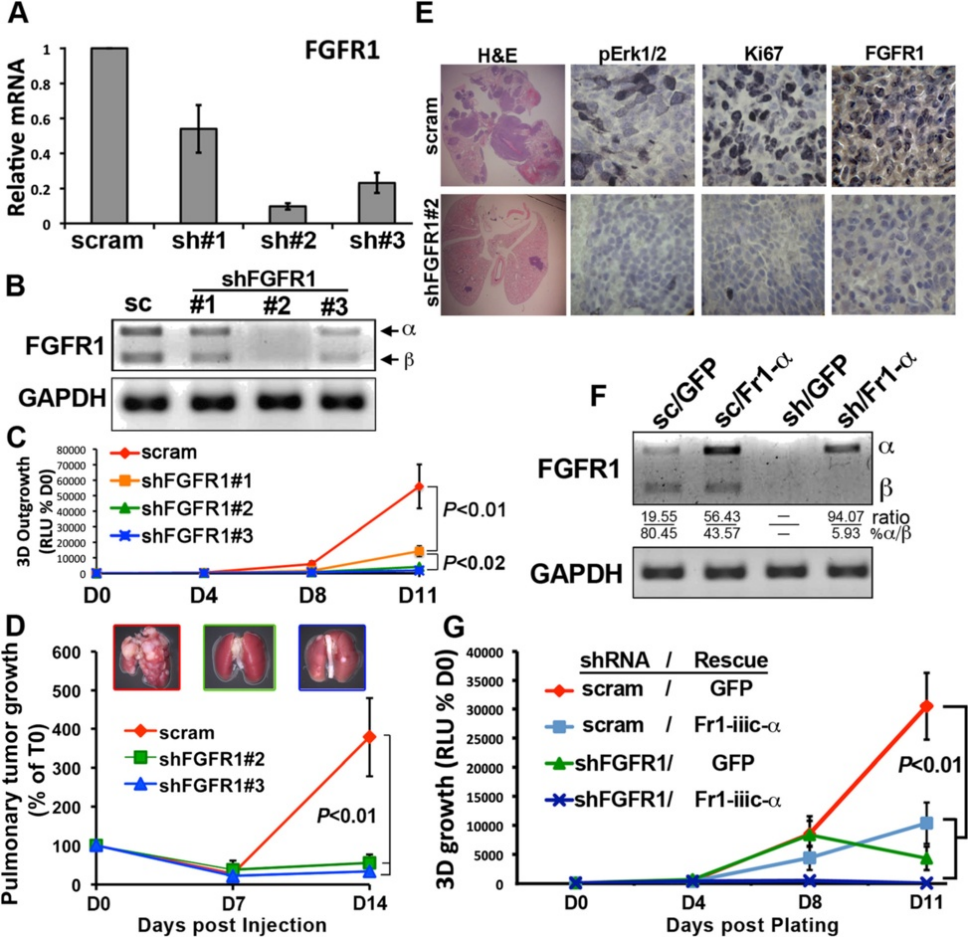
**Depletion of fibroblast growth factor receptor type 1 inhibits pulmonary tumor outgrowth. (A)** Real-time PCR analysis of fibroblast growth factor receptor type 1 (FGFR1) in D2.A1 cells expressing a nontargeting control short hairpin RNA (shRNA; scram) or three unique shRNA sequences targeting FGFR1 (sh#1 to sh#3). Data are the mean (±SD) of three independent experiments. **(B)** RT-PCR analysis of D2.A1 cells expressing the FGFR1 shRNA targeting sequences as described in (A). Primers used flank the α exon of FGFR1 and thus depict inclusion (α) or exclusion (β) of this exon. GAPDH, Glyceraldehyde 3-phosphate dehydrogenase; sc, scrambled short hairpin RNA. **(C)** Three-dimensional outgrowth of the FGFR1-depleted D2.A1 cells described in (A) and (B) was quantified by bioluminescence over the course of 11 days (D0 to D11). Data are the means (±SE) of two independent experiments completed in triplicate, resulting in the indicated *P* value. RLU, Relative light units. **(D)** D2.A1 cells expressing FGFR1 shRNAs #2 or #3 were injected into the lateral tail vein of female BALB/c mice, and pulmonary tumor formation was quantified by bioluminescence. Data are the mean (±SE) area flux values at the indicated time points expressed as a percentage of the injected value (% of T0; *P* < 0.01; n = 5 mice/group). Insets: images of representative lungs 21 days after tail vein injection with the indicated cells. **(E)** Pulmonary tissues from mice injected with control (scram) and FGFR1-depleted (shFr1#2) D2.A1 cells were stained with hematoxylin and eosin (H&E) or analyzed by immunohistochemistry for phosphorylation of extracellular signal-regulated kinases 1 and 2 (pErk1/2), the proliferation marker Ki67 or FGFR1. H&E images were taken at a 1x magnification, while all immunohistochemistry images were taken at a 400x magnification. **(F)** RT-PCR analysis of D2.A1 cells expressing a scrambled shRNA (sc) or the #2 shRNA targeting murine FGFR1 (sh). Depletion of endogenous FGFR1 in these cells was rescued by expression of a nontargeted human full-length form of FGFR1-α-IIIc (Fr1-α) or green fluorescent protein (GFP) as a control. The relative ratio of α to β FGFR1 transcripts is shown. **(G)** Three-dimensional outgrowth of the FGFR1-depleted and rescued D2.A1 cells described in (C) was quantified by bioluminescence. Data are the mean (±SE) of three independent experiments completed in triplicate, resulting in the indicated *P* value.

Our data thus far demonstrate that FGFR1 is stably upregulated following oncogenic TGF-β signaling (Figure 3A) and its stimulation of pulmonary metastasis (Figure 4B). To validate these findings in the context of metastatic outgrowth, we next took a genetic approach to specifically target FGFR1 expression in the well-established D2.A1 model of pulmonary metastatic outgrowth [2,35,36]. In accordance with the NME progression series (Figure 4B), the pulmonary outgrowth proficient D2.A1 cells readily express FGFR1-β-IIIc (Additional file 8: Figure S4). Using pan-FGFR1 primers (Figure 5A), as well as those that depict FGFR1-α and -β isoform expression (Figure 5B), we identified three unique shRNAs that elicit differential depletion of FGFR1. All of the FGFR1-targeting constructs inhibited the outgrowth of D2.A1 cells when cultured in a three dimensional (3D) matrix that recapitulates the pulmonary microenvironment (Figure 5C) [2,35-37]. Importantly, the extent to which D2.A1 organoid outgrowth was inhibited was proportional to the level of FGFR1 depletion (Figures 5A to 5C). Further *in vivo* examination of the two most effective FGFR1 targeting sequences demonstrated that depletion of FGFR1 potently blocked pulmonary tumor outgrowth (Additional file 9: Figure S5; Figure 5D). Immunohistochemical analysis of these pulmonary tumors indicated that depletion of FGFR1 was associated with decreased phosphorylation of the FGFR effectors extracellular signal-regulated protein kinases 1 and 2 (ERK1/2) and with decreased staining for the proliferation marker Ki67 (Figure 5E). Interestingly, both of the two most effective shRNA constructs elicited a compensatory increase in the expression of the α isoform of FGFR2 (Additional file 8: Figure S4). These results and previous data suggesting an antitumorigenic role for the FGFR-α isoform [15,16] led us to test the hypothesis that pulmonary metastatic outgrowth is stimulated specifically by the expression of FGFR1-β and, conversely, is inhibited by that of FGFR1-α. To this end, we rescued FGFR1-depleted D2.A1 cells (that is, shFGFR1#2) with expression of a non-targeted form of FGFR1-α-IIIc (Figure 5F). When passaged at the same density as control cells, D2.A1 cells that expressed ectopic FGFR1-α displayed a decreased rate of cell growth (Additional file 10: Figure S6).

**Figure 6:**
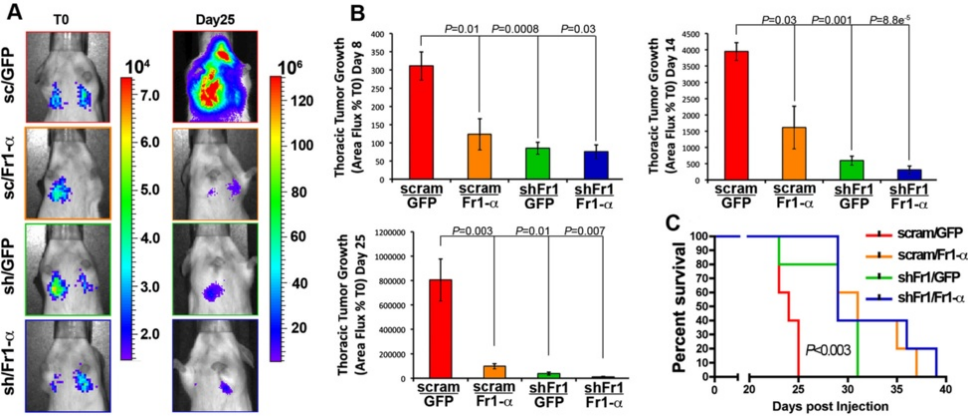
**Expression of fibroblast growth factor receptor type 1, α isoform, has a dominant inhibitory affect during pulmonary tumor formation. (A)** D2.A1 cells were constructed to express a scrambled short hairpin (shRNA; sc or scram) or the #2 shRNA targeting murine fibroblast growth factor receptor type 1 (FGFR1; sh or shFr1). Depletion of endogenous FGFR1 in these cells was rescued with a nontargeted human full-length form of the third (III) extracellular immunoglobulin (III-Ig) domain of the α isoform of FGFR1 (FGFR1-α-IIIc; Fr1-α) or green fluorescent protein (GFP) as a control. These cells (5 × 10^5^) were injected into the lateral tail vein of female BALB/c mice. Shown are representative bioluminescence images of mice from each group at the time of injection (T0) and 25 days later (day 25). **(B)** Bioluminescent quantification at the indicated time points for the cohorts described in (A). Data are the mean (±SE) thoracic area flux values normalized to the injected values, resulting in the indicated *P* values (*n* = 5 mice per group). **(C)** Survival analysis of the cohorts described in (A) resulting in at least the indicated *P* value (*n* = 5 mice per group).

FGFR1 is known to be transactivated by the extracellular matrix [38], and FGF signaling can be modulated by β3 integrin [39]. Along these lines, we previously demonstrated enhanced expression of β3 integrin in D2.A1 cells when propagated within a three-dimensional extracellular matrix as compared to traditional two-dimensional tissue culture plastic [2]. Therefore, we sought to assess the impact of FGFR1-α expression on the three-dimensional outgrowth of the D2.A1 cells. Importantly, enhanced expression of FGFR1-α inhibited the three-dimensional outgrowth of D2.A1 cells to an extent similar to that achieved by depletion of total FGFR1 (Figures 5F and 5G). Consistent with these three-dimensional organotypic outgrowth analyses, D2.A1 cells that expressed ectopic FGFR1-α also demonstrated a dramatically reduced capacity to produce pulmonary tumors in mice upon tail vein inoculation (Figures 6A and 6B), an event that significantly extended the lives of mice harboring FGFR1-α tumors (Figure 6C). Collectively, these findings indicate that the antitumorigenic nature of FGFR1-α is dominant within the pulmonary microenvironment. Moreover, these data underscore the dichotomous nature of the α and β isoforms of FGFR, a relationship that can be understood diagnostically (Figure 4D) and used to guide the application of therapeutic inhibitors against FGFR for the treatment of BC.

### Pharmacologic inhibition of FGFR kinase activity delays pulmonary outgrowth

**Figure 7:**
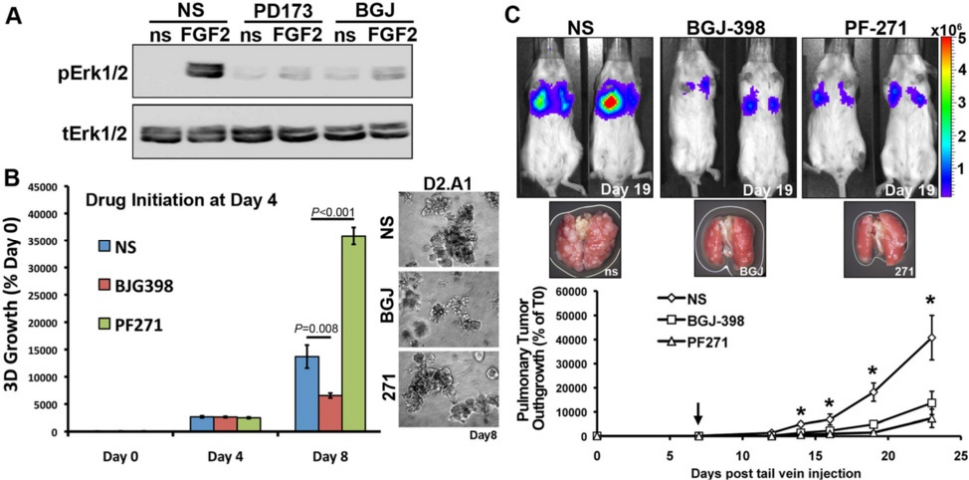
**Therapeutic targeting of fibroblast growth factor receptor kinase activity delays the outgrowth of established pulmonary tumors. (A)** Metastatic D2.A1 cells were grown under three-dimensional organotypic conditions for 4 days and not stimulated (NS) or pretreated with fibroblast growth factor receptor (FGFR) kinase inhibitor PD173074 (PD173) or BGJ398 (BGJ) for 6 hours prior to being not stimulated (ns) or stimulated with FGF2 for 30 minutes, at which point the cells were analyzed by immunoblotting for phosphorylated and total extracellular signal-regulated kinases 1 and 2 (pErk1/2 and tErk1/2, respectively). **(B)** D2.A1 tumor organoids were established for 4 days under three-dimensional culture conditions, at which point the tumor organoids were treated with BGJ398 (1 μM) or PF271 (1 μM). Data are the bioluminescence values normalized to the plated value and are the mean (±SE) of two independent experiments completed in triplicate, resulting in the indicated *P* values. Inset: Photomicrographs (100x magnification) of the D2.A1 three-dimensional tumor organoids 4 days after initiation of treatment with the indicated inhibitors. **(C)** D2.A1 cells were injected into the lateral tail vein of female BALB/c mice and allowed to establish pulmonary tumors for 7 days, at which point the mice were randomized and split into the three cohorts that received vehicle, BGJ398 or PF271 (50 mg/kg) daily via oral gavage (arrow indicates treatment initiation). Pulmonary tumor outgrowth was monitored by bioluminescence at the indicated time points (n = 5 mice per group). Bioluminescent images and ex vivo photomicrographs show the lungs from representative mice in each treatment group. Data are the mean (±SE) pulmonary bioluminescence values at the indicated time points relative to the injected value (% of D0), *P<0.05.

Given our findings thus far demonstrating the contrasting nature of FGFR1-α versus FGFR1-β in regulating pulmonary metastatic outgrowth, we next sought to assess the impact of therapeutically targeting the shared kinase domain of these FGFR isoforms. Importantly, FGF2-induced phosphorylation of ERK1/2 within tumor organoids growing in a three-dimensional matrix that recapitulates the pulmonary microenvironment could be effectively blocked using two different pharmacological inhibitors of FGFR kinase activity (Figure 7A). To model the therapeutic treatment of established pulmonary metastases, we grew D2.A1 cells for 4 days under three-dimensional organotypic culture conditions, at which point tumor organoids were treated with the clinically relevant FGFR kinase inhibitor, BGJ-398 [40]. Treatment with BGJ-398 significantly decreased the subsequent three-dimensional outgrowth of these established tumor organoids (Figure 7B). The importance of this finding is underscored by the fact that FAK1/2 kinases are known to be critically involved in the initiation of three-dimensional outgrowth [25,35]. However, treatment of these established and actively growing tumor organoids with the FAK1/2 inhibitor PF271 (PF271) actually increased their outgrowth (Figure 7B). These findings, together with those of our previous studies [2,25], highlight the critical changes that take place as outgrowth initiation is driven by a mesenchymal state, which gives way to a MET program during macroscopic pulmonary outgrowth. Importantly, the maintenance of MET-associated outgrowth requires FGFR signaling, but not FAK1/2 signaling. To verify the *in vivo* efficacy of BGJ-398 within the pulmonary microenvironment, D2.A1 cells were injected into the lateral tail vein and allowed to establish pulmonary lesions for 7 days, at which point mice bearing D2.A1 pulmonary tumors were treated daily with BGJ-398 via oral gavage. As shown in Figure 7C, administration of BGJ-398 impaired pulmonary tumor outgrowth to an extent similar to that of PF271 (Figure 7C). Taken together, these findings highlight the complexities of outgrowth initiation and maintenance and suggest that therapeutic targeting of FGFR can potently inhibit these processes.

## Discussion

EMT:MET cycles represent essential physiological processes that occur during critical points in the development, maintenance and repair of wounded epithelial tissues [41]. Through these processes, normal epithelial cells have the capacity to take on certain mesenchymal characteristics and then accurately return to their initial epithelial state. However, as has been observed in numerous other studies and quantified in our present study as shown in Figure 1, the EMT:MET process is pathologically engaged by carcinoma cells during the metastatic cascade. Therefore, in the present study, we sought to address the hypothesis that, following initiation of metastatic outgrowth, tumor cells inaccurately complete the MET program and enter into a secondary epithelial state that is similar to, but critically unique to, the epithelial characteristics of the primary tumor cells from which they are derived. Using microarray analyses, we defined numerous factors that are differentially expressed between nonmetastatic mammary tumor cells and those that have undergone a metastasis-inducing treatment with TGF-β. Overall, these findings will contribute to our understanding of the unique growth properties of metastatic lesions as compared to their corresponding primary tumors.

Using the spontaneously metastatic 4T1 model in combination with a stable E-cad-luciferase reporter system, we quantified the dynamic regulation of E-cad expression during the various steps of the metastatic cascade (Figure 1). Because these cells were derived from a spontaneous tumor, however, they almost certainly underwent one or more EMT:MET cycles during their original and natural development. Therefore, we utilized our EGFR transformation model to specifically demonstrate that TGF-β treatment is sufficient for the acquisition of metastatic properties (Figure 2). Indeed, immunohistochemical analyses and subculture of the primary tumors demonstrated that several changes in gene expression initiated *in vitro* were maintained in the primary tumor. However, *ex vivo* subculture of the resulting metastases clearly indicates that these cells have renewed expression of E-cad and enter into a secondary epithelial state. Just as with the 4T1 model, this new epithelial state easily gives way to a secondary spontaneous EMT and metastatic cycle, as secondary engraftment of these cells onto the mammary fat pad leads to robust pulmonary metastasis without TGF-β treatment prior to their inoculation into mice. Subculture of these metastases yielded the NME-LM2 cell line and established an isogenic progression series of cell lines that possess increasing metastatic potential, ranging from normal (NMuMG) to low-grade and nonmetastatic (NME) to high-grade and metastatic (NME-LM).

Using our NME progression series, various human BC cell lines and patient-derived tissue samples, we verified the stable upregulation of FGFR1-β-IIIc that we initially identified in our *in vitro* TGF-β treatment and recovery microarray analyses. As such, we further investigated the role of FGFR in facilitating late-stage metastatic tumor outgrowth. Our data clearly indicate that cellular transformation is required in conjunction with TGF-β treatment to facilitate sustained upregulation of FGFR1 (Figure 3). In this case, the means of cellular transformation is overexpression of EGFR, and we previously demonstrated that EGFR is downregulated following the *in vitro* TGF-β treatment and recovery protocol used in our present study [2]. Therefore, it is interesting to note that maintained upregulation of FGFR may serve to explain the disparity between the power of EGFR as a predictive marker of poor prognosis in BC [6] and the failure of EGFR-targeted therapies in the treatment of metastatic BC [7-9]. Studies aimed at further elucidating the role of FGFR in the inherent resistance of BCs to EGFR-targeted therapies are currently underway in our laboratory. Along these lines, we previously demonstrated that metastatic D2.A1 cells have diminished expression of EGFR compared to their nonmetastatic and isogenic D2.OR counterparts [2]. Interestingly, D2.A1 cells predominantly express FGFR1-β-IIIc, which is consistent with their metastatic phenotype. Importantly, depletion of total FGFR1 and ectopic expression of FGFR1-α-IIIc similarly inhibited pulmonary tumor outgrowth in the present study (Figure 6). Thus, our findings expand upon the results of previous studies that have linked FGFR1-β expression to the development of BC [15] and work in glioblastomas whose apoptosis was readily induced by administration of morpholino oligonucleotides to reestablish inclusion of the α exon [16].

Using flanking primer sets, we were able to identify human mammary tumor samples that not only upregulated FGFR1 expression but also aberrantly excluded the α exon as compared to their matched normal samples (patient 1 and patient 7) (Figure 4). Developing this assay as a diagnostic screening test to detect individual FGFR isoform expression could prove to be highly beneficial in prospectively identifying those patients who would most likely benefit from FGFR inhibitor therapy. Indeed, administration of BGJ-398 completely inhibited the activity of FGFR under physiologic conditions and potently delayed pulmonary tumor outgrowth of the D2.A1 cells. These data are consistent with the fact that the D2.A1 cells primarily express FGFR1-β. However, given our genetic studies identifying the antitumorigenic nature of FGFR1-α, administration of FGFR inhibitor therapies to patients whose tumors express this isoform (patients 2 and 13) (Figure 4) could potentially prove to be detrimental. In fact, this pro- and antitumorigenic dichotomy between FGFR isoforms likely contributes to the limited efficacy of BGJ-398 in inhibiting the outgrowth of the D2.A1 cells, as these cells do endogenously express detectable levels of the FGFR1-α isoform. In contrast to this cell culture model, the breast tumor samples of two patients analyzed in our present study (patients 1 and 13) yielded very clear upregulation of one isoform or the other, again supporting the use of this FGFR diagnostic approach as a predictive biomarker for initiation of FGFR inhibitor therapy.

## Conclusion

Our studies demonstrate the dynamics of EMT and MET as BC progresses from carcinoma *in situ* to full-blown metastatic disease. FGFR and several other factors identified herein represent a signature of oncogenic TGF-β signaling that does not return to baseline during recovery from ligand exposure. This failure to accurately execute the MET process sets the stage for FGFR to act as a potent driver of pulmonary metastatic outgrowth, even if it may not have been an initiator of primary tumor formation. Overall, our findings have important implications related to the means by which science and medicine undertake targeting of FGFR for the treatment of metastatic BC.

## Abbreviations

BC: Breast cancer; CTC: Circulating tumor cell; E-cad: Epithelial cadherin; EGFR: Epidermal growth factor receptor; EMT: Epithelial–mesenchymal transition; ER-α: Estrogen receptor α; FGFR: Fibroblast growth factor receptor; MET: Mesenchymal–epithelial transition; TGF-β: Transforming growth factor β.

## Competing interests

The authors declare that they have no competing interests.

## Authors’ contributions

MW carried out the molecular, cellular and *in vivo* studies, conceptualized the experiments and drafted the manuscript. BS, KSA and MT carried out molecular and cellular studies and critically revised the manuscript for important intellectual content. WS helped conceptualize the experiments and draft the manuscript. All authors made substantial contributions to the design of the study. All authors read and approved the final version of the manuscript.

## Supplementary Material

Additional file 1: Table S1 Table listing the sequences of the oligos used for the indicated applications. Also listed are the sequences of the fibroblast growth factor receptor type 1 (FGFR1)–targeted short hairpin RNA (shRNAs). Click here for file

Additional file 2: Table S2 Table listing genes that were regulated more than threefold between untreated normal mammary epithelial (NME) cells (before transforming growth factor treatment; pre-TGF) and those that had transitioned through a cycle of epithelial–mesenchymal transition (EMT) and mesenchymal–epithelial transition (MET) (EMT:MET) (post-TGF-Rec) as described in the Methods section of the text. As shown, microarray analyses were conducted in triplicate, resulting in the indicated averages and calculated fold changes in expression. The complete microarray data set can be found in the Gene Expression Omnibus database [GEO:GSE54491]. Click here for file

Additional file 3: Table S3 Table listing the antibodies used for the indicated applications, the dilution at which they were used and the supplier information. Click here for file

Additional file 4: Figure S1 Transforming growth factor β (TGF-β)–induced normal mammary epithelial (NME) cell metastases are highly aggressive upon secondary fat pad inoculation. (A) Following primary tumor removal, untreated (Pre-TGF) and TGF-β-treated (Post-TGF) NME primary tumors were disassociated and subcultured in the presence of hygromycin (500 μg/ml). The resultant cultures were grown on two-dimensional standard tissue culture plastic (2D-plastic) or under three-dimensional organotypic culture conditions (3D culture). Shown are representative phase contrast images (original magnification, 200×) depicting the typical growth morphologies of these *ex vivo* tumor cells. **(B) ***Ex vivo* tumors derived from TGF-β-treated and untreated tumors were disassociated and analyzed by flow cytometry for expression of epithelial cadherin (E-cad). **(C)** Parental NME cells and their TGF-β-induced lung metastatic derivatives (NME-LM; 1 × 10^6^ cells/mouse) were engrafted onto the mammary fat pad of *nu*/*nu* mice, and primary tumor growth and recurrence were monitored by digital caliper measurements (*n* = 5 mice per group). Click here for file

Additional file 5: Figure S2 Transforming growth factor β treatment and metastatic subculture select for cells with an epithelial phenotype but enhanced metastatic potential. (A) Schematic representation and phase contrast photomicrographs of the normal mammary epithelial (NME) cell progression series. **(B)** Following transforming growth factor β (TGF-β)–induced metastasis, several markers of epithelial–mesenchymal transition (EMT) (epithelial cadherin (E-cad), vimentin, and neuronal cadherin (N-cadherin)) in the NME lung metastatic (NME-LM) cells returned to levels comparable to those of the parental NME cells. **(C)** RT-PCR analysis of E-cad showing similar levels across the NME progression series. Data in (B) and (C) are representative of two independent experiments yielding similar results. Click here for file

Additional file 6: Figure S3 Autocrine expression of epidermal growth factor receptor ligands are downregulated following transforming growth factor β treatment and recovery. Normal mammary epithelial (NME) cells were stimulated with transforming growth factor β1 (TGF-β1; 5 ng/ml) and allowed to recover as detailed in the Methods section of the text. RNA was gathered, and global gene expression was assessed by microarray analysis (Supplementary Table S1). Real-time PCR was carried out to confirm downregulation of the epidermal growth factor receptor (EGFR) ligands betacellulin, amphiregulin, and epiregulin. Numbers indicate fold downregulation for each gene. Click here for file

Additional file 7: Table S4 Fibroblast growth factor receptor expression is increased in breast cancer tumors as compared to matched normal tissues. Twenty breast cancer (BC) tumor biopsies were analyzed by RT-PCR for expression of fibroblast growth factor receptor (FGFR) types 1 to 4, whose expression levels were normalized against RNA gathered from adjacent normal tissues. Data shown are the mean (±SE) fold increases in FGFR levels as compared to the matched normal tissue. Click here for file

Additional file 8: Figure S4 Genetic depletion of fibroblast growth factor receptor type 1 abrogates pulmonary tumor formation in mice. Bioluminescent images of representative mice injected with D2.A1 cells expressing a control (scrambled short hairpin RNA; scram) or two unique fibroblast growth factor receptor type 1 (FGFR1)–targeting short hairpin RNAs (shFr1#2 and shFr1#3). Images were taken at the time of injection (time 0; T0) and 3 weeks following injection. Click here for file

Additional file 9: Figure S5 Genetic depletion of fibroblast growth factor receptor type 1 results in a compensatory increase in fibroblast growth factor receptor type 2, α isoform, expression. (A) Real-time PCR analysis of fibroblast growth factor receptor types 2 to 4 (FGFR2 to FGFR4) in D2.A1 cells expressing a nontargeting control scrambled short hairpin RNA (scram) or two unique short hairpin RNA (shRNA) sequences targeting FGFR1 (shFGFR1#2 and shFGFR1#3). Data are the mean (±SD) of four independent experiments. **(B)** RT-PCR analyses of cells expressing FGFR1 shRNAs as described in (A) using isoform-specific primer sets for FGFR1 and FGFR2 as detailed in Figure 4A in the main text. Click here for file

Additional file 10: Figure S6 Expression of third extracellular immunoglobulin domain of fibroblast growth factor receptor type 1, isoform α, decreases the *in vitro* growth of D2.A1 cells. D2.A1 cells expressing a control scrambled short hairpin RNA (shRNA; scram) or a fibroblast growth factor receptor type 1 (FGFR1)–specific shRNA (shFGFR1) were transduced with retroviral particles harboring transcripts of either green fluorescent protein (GFP) or the third (III) extracellular immunoglobulin (III-Ig) domain of fibroblast growth factor receptor type 1, isoform α (FGFR1-α-IIIc). These populations were selected with G418 and cultured at identical cell densities for an additional three passages (P1 to P3). Click here for file
