# Supplementary material for: Fibroblast growth factor receptor splice variants are stable markers of oncogenic transforming growth factor β1 signaling in metastatic breast cancers
Source: Breast Cancer Res. 2014 Mar 11;16(2):R24. doi: 10.1186/bcr3623 (PMC4053226; doi:10.1186/bcr3623)
Supplement: Additional file 1: Table S1 — Table listing the sequences of the oligos used for the indicated applications. Also listed are the sequences of the fibroblast growth factor receptor type 1 (FGFR1)–targeted short hairpin RNA (shRNAs). [file bcr3623-S1.pdf]

Table S1

Target	Application	Sequence (5' to 3')
GAPDH	Real Time PCR-Sense	5'-CAACTTTGGCATTGTGGAAGGGCTC
GAPDH	Real Time PCR-Antisense	5'-GCAGGGATGATGTTCTGGGCAGC
hFGFR1	Real Time PCR-Sense	5'- CGCCCCTGTACCTGGAGATCATCA
hFGFR1	Real Time PCR- Antisense	5'- TTGGTACCACTCTTCATCTT
hFGFR2	Real Time PCR-Sense	5'- GCCTGGAAGAGAAAAGGAGATTAC
hFGFR2	Real Time PCR-Antisense	5'- GGATGACTGTTACCACCATACA
hFGFR3	Real Time PCR-Sense	5'- CTCGCGCTCTGCGTGGCCGT
hFGFR3	Real Time PCR-Antisense	5'- TTCTTGTCCATCCGCTCGGG
hFGFR4	Real Time PCR-Sense	5'- GATGGACAGGCCTTTCATGGG
hFGFR4	Real Time PCR-Antisense	5'- TGCTGCGGTCCATGTGGGGTCCTC
mFGFR1	Real Time PCR-Sense	5'- CACCGCTCTACCTGGAGATCATTA
mFGFR1	Real Time and RT PCR-Antisense	5'- TTGGTGCCGCTCTTCATCTT
mFGFR2	Real Time PCR-Sense	5'- GCCTGTGAGAGAGAAGGAGATCAC
mFGFR2	Real Time and RT PCR-Antisense	5'- AGATGACTGTCACCACCATGCA
mFGFR3	Real Time PCR-Sense	5'- CTAGTGTTCTGCGTGGCGGT
mFGFR3	Real Time PCR-Antisense	5'- TTCTTATCCATTCGCTCCGG
mFGFR4	Real Time PCR-Sense	5'- CTGTTGAGCATCTTTCAGGG
mFGFR4	Real Time PCR-Antisense	5'- CGTGGAAGGCCTGTCCATCC
mFGFR1-iiib	RT-PCR-Sense	5'- CGGGAATTAATAGCTCGGATGC
mFGFR1-iiic	RT-PCR-Sense	5'- GGAGTTAATACCACCGACAAA
mFGFR2-iiib	RT-PCR-Sense	5'- CACTCGGGGATAAATAGCTCC
mFGFR2-iiic	RT-PCR-Sense	5'- CGGTGTTAACACCACGGAC
mFGFR1-α/β	RT-PCR-Sense	5'- TTCTGGGCTGTGCTGGTCAC

mFGFR1-α/β	RT-PCR-Antisense	5'- GCGAACCTTGTAGCCTCCAA
mFGFR2-α/β	RT-PCR-Sense	5'- TTCATCTGCCTGGTCTTGGT
mFGFR2-α/β	RT-PCR-Antisense	5'- AATAAGGCTCCAGTGCTGGTTTC
mAREG	Real Time PCR-Sense	5'- CAGGGGACTACGACTACTCAGA
mAREG	Real Time PCR-Antisense	5'- GATAACGATGCCGATGCCAATA
mBTC	Real Time PCR-Sense	5'- GCATTACTGCATCCATGGGAG
mBTC	Real Time PCR-Antisense	5'- GCTTGCCACCAGCTTGTGATA
mEREG	Real Time PCR-Sense	5'- GGAATTCTGACGCTGCTTTGTCTAGGTT
mEREG	Real Time PCR-Antisense	5'- CAAGCTTTATGCATCCAGCGGTTATGAT
mFGFR1	shRNA#1	CCGG*CTTGCCTCTAAGAAGTGTATA***CTCGAG***TATACACTTCTTAGAGGCAAG*TTTTT
mFGFR1	shRNA#2	CCGG*CCTGGAGCATCATAATGGATT***CTCGAG***AATCCATTATGATGCTCCAGGTTTTT*
mFGFR1	shRNA#3	CCGG*CGAGGATAACGTAATGAAGAT***CTCGAG***ATCTTCATTACGTTATCCTCG*TTTTT

Underlined text denotes complimentary RNA-targeted sequence, while **bold text** denotes hairpin sequence.
