# Supplementary material for: Fibroblast growth factor receptor splice variants are stable markers of oncogenic transforming growth factor β1 signaling in metastatic breast cancers
Source: Breast Cancer Res. 2014 Mar 11;16(2):R24. doi: 10.1186/bcr3623 (PMC4053226; doi:10.1186/bcr3623)
Supplement: Additional file 3: Table S3 — Table listing the antibodies used for the indicated applications, the dilution at which they were used and the supplier information. [file bcr3623-S3.pdf]

Table S3

Antibody	Dilution	Supplier (Catalogue #)
E-Cadherin	1:5000	BD Biosciences (610182)
β3 Integrin	1:1000	Cell Signaling Technologies (4702)
β1 Integrin	1:1000	Cell Signaling Technologies (4706)
EGFR	1:1000	Cell Signaling Technologies (2646)
EGFR1 for flow cytometry	1:50	Harlan Laboratories (custom)
Total-Erk1/2	1:2000	Cell Signaling Technologies (4695)
β-actin	1:1000	Santa Cruz Biotechnologies (sc-1616)
ER-α	1:1000 for Immunoblot 1:100 for IHC	Santa Cruz Biotechnologies (sc-542)
Fibronectin	1:2000 for Immunoblot 1:100 for IHC	BD Biosciences (610077)
FGFR1	1:100 for IHC	Santa Cruz Biotechnoligies (sc-121)
Phospho-Erk1/2	1:2000 for Immunoblot 1:100 for IHC	Cell Signaling Technologies (9101)
