# Supplementary figures and images for: Fibroblast growth factor receptor splice variants are stable markers of oncogenic transforming growth factor β1 signaling in metastatic breast cancers
Source: Breast Cancer Res. 2014 Mar 11;16(2):R24. doi: 10.1186/bcr3623 (PMC4053226; doi:10.1186/bcr3623)

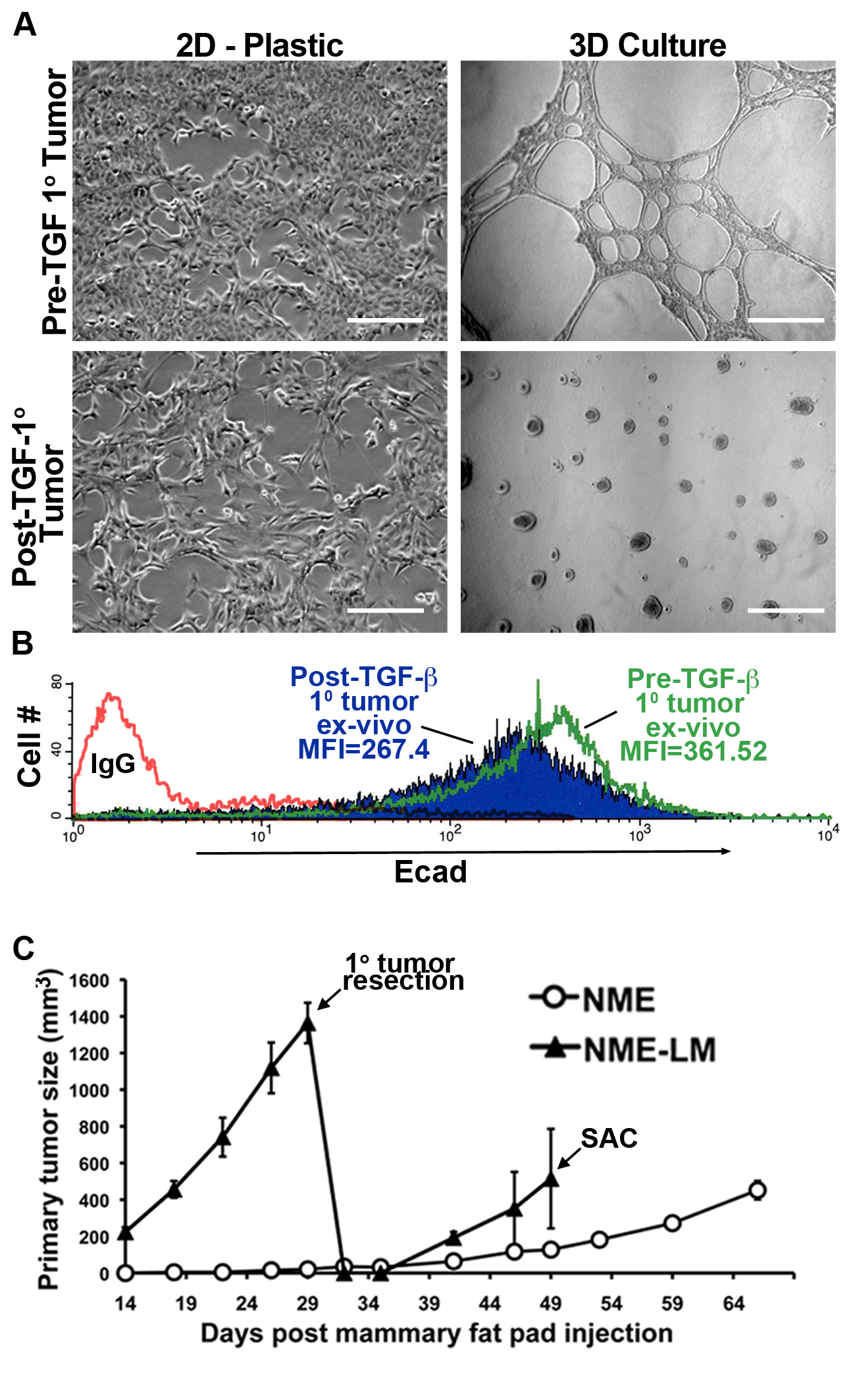

Supplement: Additional file 4: Figure S1 — Transforming growth factor β (TGF-β)–induced normal mammary epithelial (NME) cell metastases are highly aggressive upon secondary fat pad inoculation. (A) Following primary tumor removal, untreated (Pre-TGF) and TGF-β-treated (Post-TGF) NME primary tumors were disassociated and subcultured in the presence of hygromycin (500 μg/ml). The resultant cultures were grown on two-dimensional standard tissue culture plastic (2D-plastic) or under three-dimensional organotypic culture conditions (3D culture). Shown are representative phase contrast images (original magnification, 200×) depicting the typical growth morphologies of these ex vivo tumor cells. (B) Ex vivo tumors derived from TGF-β-treated and untreated tumors were disassociated and analyzed by flow cytometry for expression of epithelial cadherin (E-cad). (C) Parental NME cells and their TGF-β-induced lung metastatic derivatives (NME-LM; 1 × 10^6^ cells/mouse) were engrafted onto the mammary fat pad of nu/nu mice, and primary tumor growth and recurrence were monitored by digital caliper measurements (n = 5 mice per group). [file bcr3623-S4.jpeg]

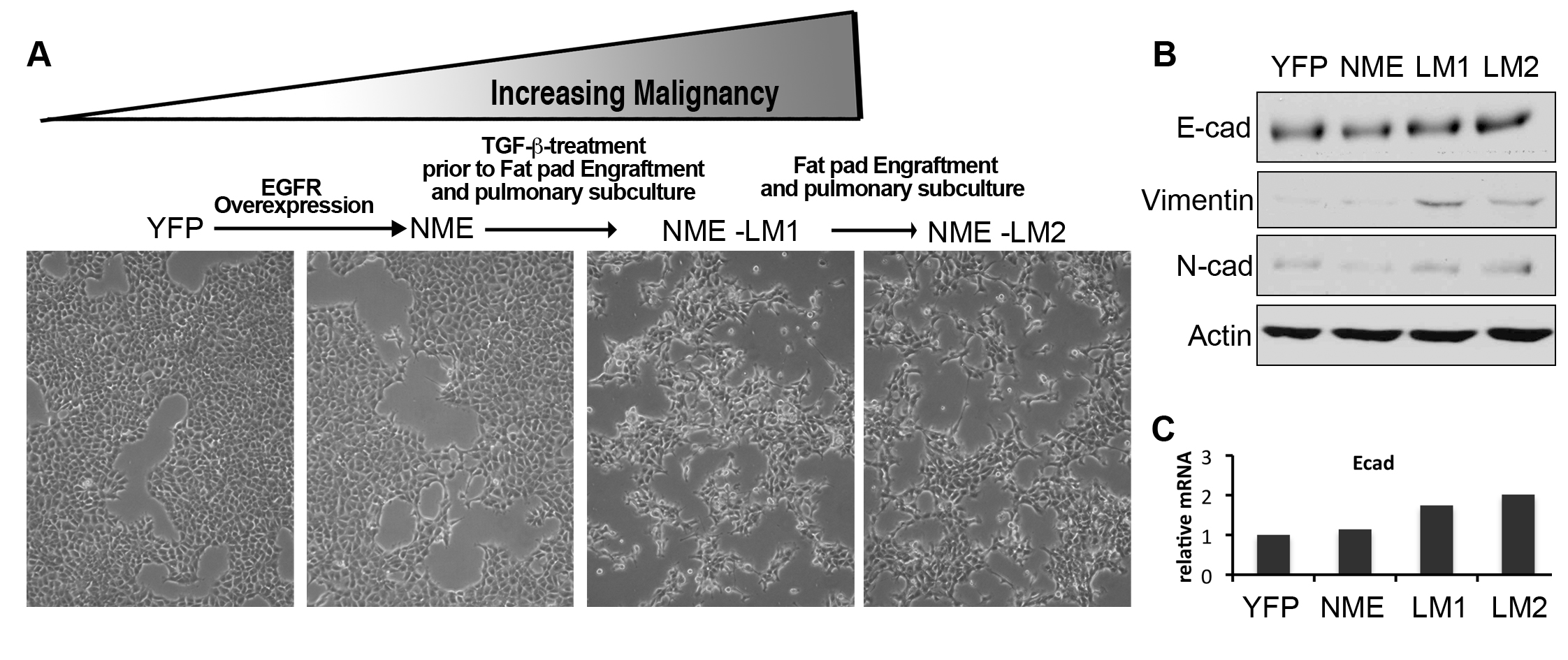

Supplement: Additional file 5: Figure S2 — Transforming growth factor β treatment and metastatic subculture select for cells with an epithelial phenotype but enhanced metastatic potential. (A) Schematic representation and phase contrast photomicrographs of the normal mammary epithelial (NME) cell progression series. (B) Following transforming growth factor β (TGF-β)–induced metastasis, several markers of epithelial–mesenchymal transition (EMT) (epithelial cadherin (E-cad), vimentin, and neuronal cadherin (N-cadherin)) in the NME lung metastatic (NME-LM) cells returned to levels comparable to those of the parental NME cells. (C) RT-PCR analysis of E-cad showing similar levels across the NME progression series. Data in (B) and (C) are representative of two independent experiments yielding similar results. [file bcr3623-S5.jpeg]

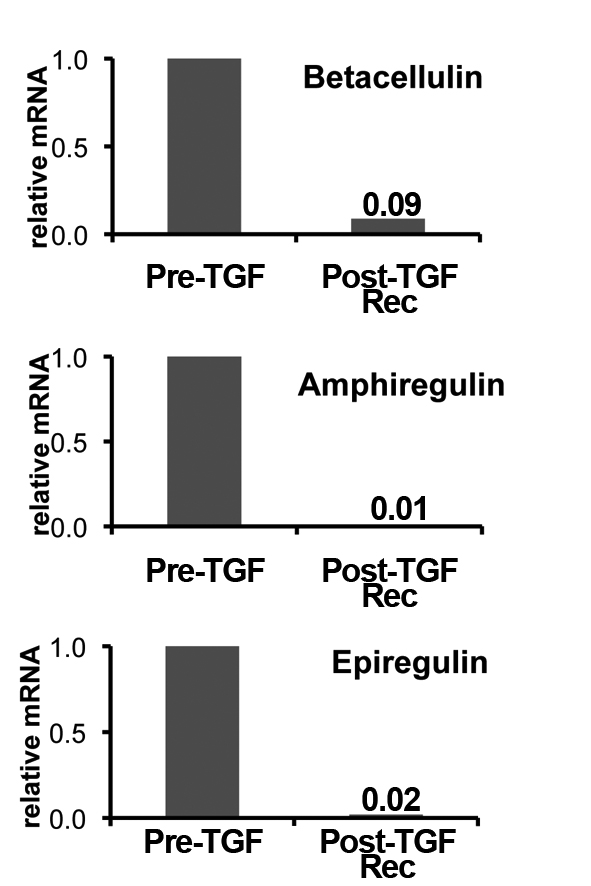

Supplement: Additional file 6: Figure S3 — Autocrine expression of epidermal growth factor receptor ligands are downregulated following transforming growth factor β treatment and recovery. Normal mammary epithelial (NME) cells were stimulated with transforming growth factor β1 (TGF-β1; 5 ng/ml) and allowed to recover as detailed in the Methods section of the text. RNA was gathered, and global gene expression was assessed by microarray analysis (Supplementary Table S1). Real-time PCR was carried out to confirm downregulation of the epidermal growth factor receptor (EGFR) ligands betacellulin, amphiregulin, and epiregulin. Numbers indicate fold downregulation for each gene. [file bcr3623-S6.jpeg]

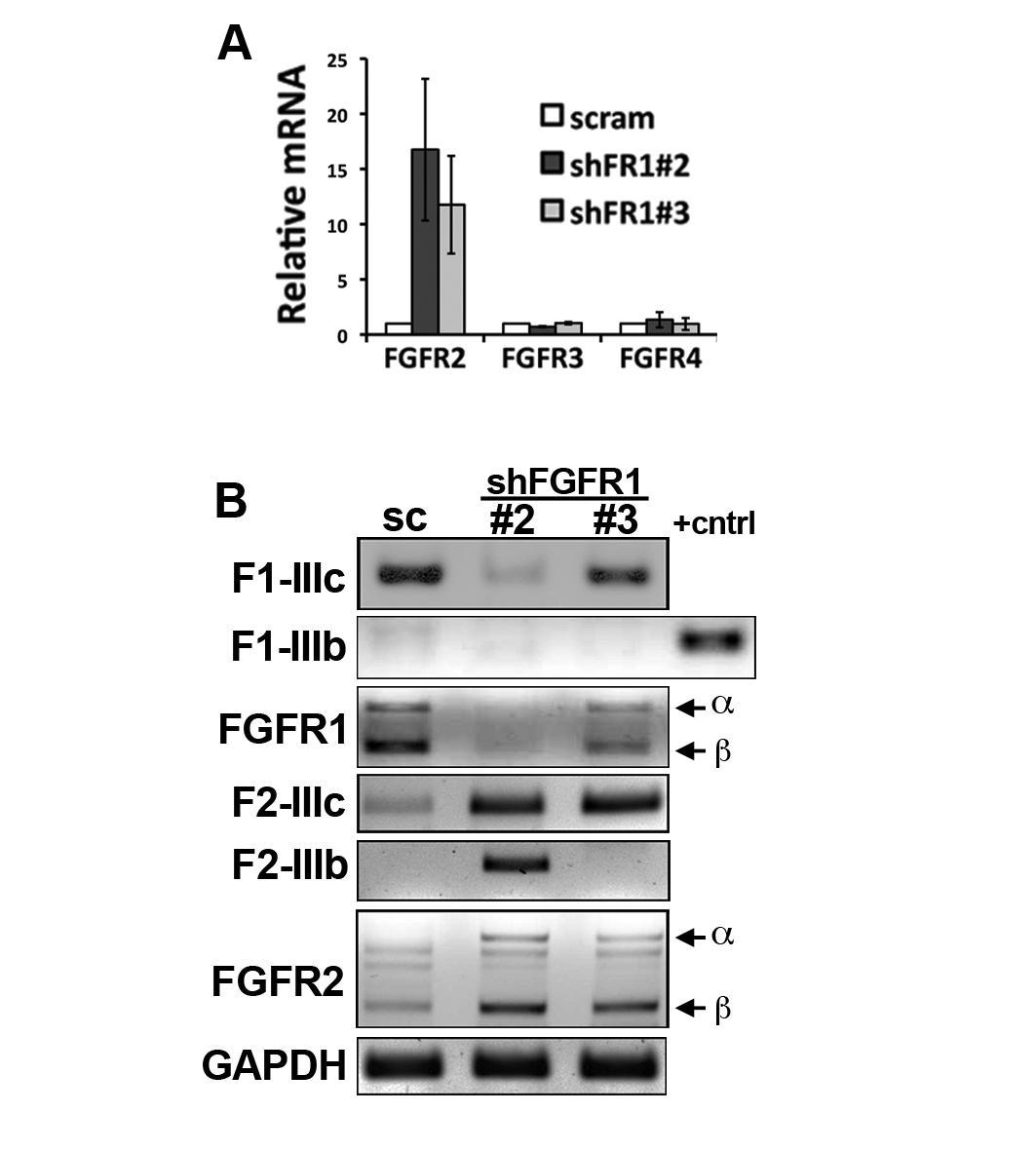

Supplement: Additional file 8: Figure S4 — Genetic depletion of fibroblast growth factor receptor type 1 abrogates pulmonary tumor formation in mice. Bioluminescent images of representative mice injected with D2.A1 cells expressing a control (scrambled short hairpin RNA; scram) or two unique fibroblast growth factor receptor type 1 (FGFR1)–targeting short hairpin RNAs (shFr1#2 and shFr1#3). Images were taken at the time of injection (time 0; T0) and 3 weeks following injection. [file bcr3623-S8.jpeg]

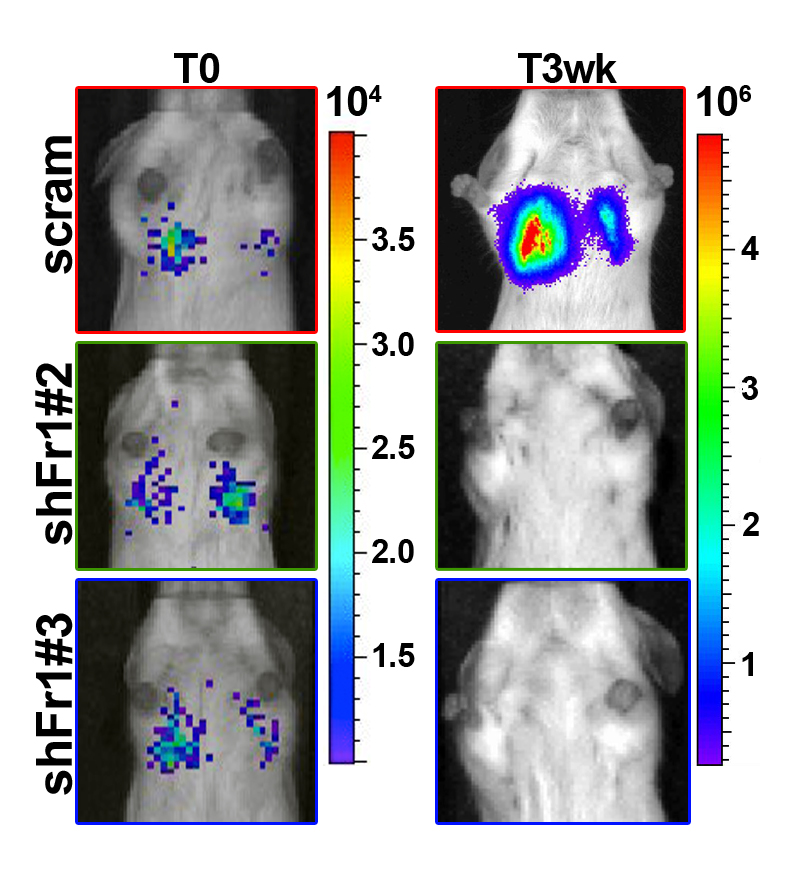

Supplement: Additional file 9: Figure S5 — Genetic depletion of fibroblast growth factor receptor type 1 results in a compensatory increase in fibroblast growth factor receptor type 2, α isoform, expression. (A) Real-time PCR analysis of fibroblast growth factor receptor types 2 to 4 (FGFR2 to FGFR4) in D2.A1 cells expressing a nontargeting control scrambled short hairpin RNA (scram) or two unique short hairpin RNA (shRNA) sequences targeting FGFR1 (shFGFR1#2 and shFGFR1#3). Data are the mean (±SD) of four independent experiments. (B) RT-PCR analyses of cells expressing FGFR1 shRNAs as described in (A) using isoform-specific primer sets for FGFR1 and FGFR2 as detailed in Figure 4A in the main text. [file bcr3623-S9.jpeg]
