# Supplementary material for: Fibroblast growth factor receptor splice variants are stable markers of oncogenic transforming growth factor β1 signaling in metastatic breast cancers
Source: Breast Cancer Res. 2014 Mar 11;16(2):R24. doi: 10.1186/bcr3623 (PMC4053226; doi:10.1186/bcr3623)
Supplement: Additional file 7: Table S4 — Fibroblast growth factor receptor expression is increased in breast cancer tumors as compared to matched normal tissues. Twenty breast cancer (BC) tumor biopsies were analyzed by RT-PCR for expression of fibroblast growth factor receptor (FGFR) types 1 to 4, whose expression levels were normalized against RNA gathered from adjacent normal tissues. Data shown are the mean (±SE) fold increases in FGFR levels as compared to the matched normal tissue. [file bcr3623-S7.pdf]

Table S4

	FGFR1	FGFR2	FGFR3	FGFR4
**Fold Regulation (+/- SE) n=20**	7.96(+/- 2.87)	8.06(+/- 2.86)	6.77(+/- 3.20)	7.75(+/- 3.10)

Supplemental Table 2. FGFR expression is increased in BC tumors as compared to matched normal tissues. Twenty BC tumor biopsies were analyzed by RT-PCR for expression of FGFRs 1-4 whose expression levels were normalized against RNA gathered from adjacent normal tissues. Data shown are the mean-fold increases (+SE) in FGFR levels as compared to the matched normal tissue.
